# MiR-920 promotes osteogenic differentiation of human bone mesenchymal stem cells by targeting HOXA7

**DOI:** 10.1186/s13018-020-01775-7

**Published:** 2020-07-10

**Authors:** Jun-pu Zha, Xiao-qing Wang, Jun Di

**Affiliations:** Department of Orthopaedics, The Third Hospital of HeBei Medical University, 139 Ziqiang Road, Shijiazhuang, 050051 China

**Keywords:** MiR-920, Human bone mesenchymal stem cells, Osteogenic differentiation, HOXA7

## Abstract

**Background:**

To explore the effect of miR-920 on osteogenic differentiation of human bone mesenchymal stem cells (hBMSCs) and the possible mechanism.

**Methods:**

Osteoporosis (OP) and healthy control bone tissues were collected, and the relative expression of miR-920 and HOXA7 was measured. hBMSCs were isolated and cultured in vitro. Alkaline phosphatase activity and miR-920 and HOXA7 relative expression were measured during osteogenic differentiation of hBMSCs. Then, bioinformatic analysis was performed to assess the potential mechanism of miR-920. MiR-920 mimic and inhibitor were introduced into hBMSCs by lipofection transfection and were used to investigate the effect of miR-920 on the osteogenic differentiation of hBMSCs. A dual luciferase reporter assay was used to identify whether the 3′UTR of HOXA7 mRNA was a direct target of miR-920. Western blotting was performed to assess whether miR-920 affected the MAPK signaling pathway.

**Results:**

We found that miR-920 was downregulated in OP patients compared with controls, while HOXA7 was upregulated, and miR-920 had a negative correlation with HOXA7 (*r* = − 0.859, *P* = 0.001). Moreover, miR-920 was increased during osteogenic differentiation of hBMSCs, while HOXA7 had the opposite tendency. Bioinformatic analysis revealed that there were a total of 207 target genes, and MAPK was a potential targeted signaling pathway. MiR-920 mimic significantly increased ALP activity, calcium deposition, osteoblastic protein expression (ALP and OSX), and p-p38 and p-JNK protein levels.

**Conclusion:**

Overall, miR-920 promotes osteogenic differentiation of hBMSCs by targeting HOXA7 through the MAPK signaling pathway.

## Background

Human bone mesenchymal stem cells (hBMSCs) are pluripotent stem cells located in the mesoderm that can be differentiated into adipocytes, osteoblasts, chondrocytes, muscle cells, and other cell types under specific conditions [1]. hBMSCs were initially isolated and identified by Friedenstein et al. [2] from bone marrow and later found to be widely present in fat, muscle, blood, and other connective tissues [3]. These cells can also inhibit the proliferation of B cells and T cells through direct interactions with cells and the cytokines produced, thus inhibiting the immune response; consequently, there is no immune rejection in clinical practice [4].

Derived from bone marrow, mesenchymal stem cells are easy to culture in vitro and can be differentiated into osteoblasts under osteogenic induction conditions, so currently, they have become ideal seed cells for bone tissue engineering, playing a great role in promoting bone repair and reconstruction in patients with osteoporosis (OP) [5]. Improving the osteogenic differentiation ability of bone marrow mesenchymal stem cells is the key to bone regeneration.

MicroRNAs (miRNAs) are a class of endogenous noncoding small RNAs with a length of 21–25 bp that were discovered in recent years [6]. They degrade target mRNA or inhibit translation by binding to target mRNA, thus playing an important regulatory role in various biological processes of cells, such as cell proliferation, apoptosis, differentiation, and aging [7, 8]. It is predicted that at least 1000 miRNAs exist in the human genome, regulating tens of thousands of protein-coding genes [9]. In this study, we collected bone tissues from OP patients and healthy controls. We found that miR-920 was decreased in OP patients.

Therefore, it is speculated that miR-920 may promote the differentiation of hBMSCs into osteoblasts by targeting HOXA7 expression. We used bioinformatic analysis to further analyze the potential mechanism of miR-920.

## Material and methods

### Reagents and instruments

The following reagents were used in this study: α-MEM medium, fetal bovine serum (FBS), penicillin, streptomycin (Gibco, USA); Percoll separation solution, RNAiso for small RNA (Wuhan Kehaojia Biotechnology Co., LTD.); dexamethasone, β-glycerophosphate, vitamin C (Sigma, USA); U6 (Shanghai Nuolun Biomedical Technology Co., LTD.); TRIzol kit, One-Step PrimeScript® miRNA cDNA Synthesis Kit, Lipofectamine 2000 transfection kit, Dual-Luciferase Reporter System (GeneCopoeia, USA); SYBR ® Premix Ex Taq II, GeneTailor Site–Directed Mutagenesis System kit (Invitrogen companies in the USA); and alkaline phosphatase activity assay kit (Nanjing Jincheng Biological Products Co., LTD.). MiR-920 mimics, inhibitor, and negative control were purchased from Shanghai Zhi Zhong Laboratory Equipment Co., Ltd. Antibodies against HOXA7, ALP, OSX, p38, p-p38, JNK, p-pJNK, and GAPDH and horseradish-peroxidase-labeled secondary antibodies were all purchased from Abcam (Cambridge, UK).

### hBMSC isolation and osteogenic differentiation

HBMSCs were isolated and cultured as previously described [10]. Generally, hBMSCs were separated from bone marrow by density gradient centrifugation. In addition, hBMSCs were seeded into flasks and cultured in an incubator at 37 °C, 5% CO_2_, and 100% relative humidity. The medium was changed every 3 days. The osteogenic induction medium (OIM) was as follows: α-MEM containing 10% FBS, 0.1 mol/L dexamethasone, 10 mmol/L β-sodium glycerophosphate, and 50 mg/L vitamin C. This study was approved by the ethics committee of the Third Hospital of HeBei Medical University (Shijiazhuang, China), and written informed consent forms were signed by all included patients.

### hBMSC transfection

MiR-920 mimic, inhibitor, and corresponding negative control were purchased from GeneChem (Shanghai, China). The sequences were as follows: miR-920 mimic NC: 5′-CUUCAGCCCUUUCUAAUCUUUAUA-3′; miR-920 mimic: 5′-AAACCGUUGCCUGCCCUCCUAAAU-3′; miR-920 inhibitor NC: 5′-CGCATTGAUUACGTAGCCTAAGCT-3′; and miR-920 inhibitor: 5′-UCACACUTAAGATGGATTGGGUUT-3′.

According to the manufacturer’s instructions, at initial osteogenic differentiation, hBMSCs were incubated with miR-920 mimic, inhibitor, and negative control at a final concentration of 30 nM by using Lipofectamine® 2000 (Invitrogen) and then incubated with osteogenic induction medium for 14 days.

### ALP and ARS

For ALP staining assays, the BCIP/NBT Alkaline Phosphatase Color Development Kit (Beyotime, Shanghai, China) was used according to the manufacturer’s instructions. According to the instructions of the alkaline phosphatase activity assay kit, alkaline phosphatase activity was quantified with a yellow liquid substrate system of alkaline phosphatase by ELISA. The alkaline phosphatase activity was standardized by collecting the total protein of cells. The absorbance value at a wavelength of 405 nm was measured by an ultraviolet spectrophotometer, and the alkaline phosphatase activity was calculated according to the following formula: alkaline phosphatase activity (U/g) = [(determination of tube absorbance value/standard tube absorbance value) × standard tube p-nitrophenol quantity]/total protein grams. For ARS staining and quantification, cells were cultured in OIM for 14 days. First, hBMSCs were fixed in 4% paraformaldehyde and then stained with 0.2% ARS (pH 4.3) for 15 min at room temperature. To remove nonspecific staining, the stained cells were washed three times with PBS. Subsequently, the calcium nodules were observed under a microscope and imaged. The calcium nodules were solubilized with 10% cetylpyridinium chloride (CPC) to quantify matrix mineralization for 30 min. To calculate calcium concentrations, the absorbance was determined at 562 nm.

### Quantitative real-time PCR (qRT-PCR)

Cells induced for 0, 3, 7, 14, and 28 days were collected and washed with PBS 3 times. RNA was extracted by TRIzol as described previously [11]. The RNA concentration was adjusted to 1 g/L with RNase-free water. Then, according to the One-Step PrimeScript ® miRNA cDNA Kit instructions, cDNA reverse transcription was performed with 4-fold diluted cDNA. Real-time quantitative PCR was performed with SYBR ® Premix Ex Taq II on a Chromo4 thermocycler. The internal control gene U6 and the Opticon-3 software were used to analyze the results. All the RT-PCRs were performed in triplicates, and the primers used for PCR are listed in Table 1.

**Table 1:** Real-time PCR primers for amplification of specific hBMSCs mRNA

Gene name	Forward primer (5′-3′)	Reverse primer (5′-3′)
HOXA7	CTTATACAATGTCAACAGCC	TCCTTATGCTCTTTCTTCC
miR-920	GCCTTCGCTCAACTGAATTG	CTCAACTGGTGTCGTGGAGTC
U6	CTTCGGCAGCACATATACT	AAAATATGGAACGCTTCACG
GAPDH	CCACTCCTCCACCTTTGAC	ACCCTGTTGCTGTAGCCA

### Western blot

Total protein was extracted from hBMSCs transfected with miR-920 mimic, inhibitor, or NC using RIPA buffer and protease inhibitors at a volume ratio of 100:1. Then, the same amount of protein was separated by sodium dodecyl sulfate-polyacrylamide gel electrophoresis (SDS-PAGE). Then, the proteins were transferred to cellulose nitrate membranes and washed with TBST with 5% skim milk at room temperature for 1 h. Next, the membranes were incubated with the following primary antibodies: anti-HOXA7 (1:2000, ProteinTech), anti-ALP (1:1000, Abcam), anti-OSX (1:1000, Abcam), anti-p-p38 (1:1000, Cell Signaling Technology), anti-p38 (1:1000, Cell Signaling Technology), anti-p-JNK (1:1000, Cell Signaling Technology), anti-JNK (1:1000, Cell Signaling Technology), and anti-GAPDH (1:3000, ProteinTech) at 4 °C overnight. The membranes were warmed up to room temperature, washed with TBST 3 times, incubated with secondary antibody (1:5000; ProteinTech) for 2 h, washed with TBST 3 times, and then ECL chemiluminescence and development were performed in the dark. Amersham Imager 600 (GE Healthcare) was used to observe the bands on the PVDF membrane.

### Luciferase assay

The PCR amplification product of the HOXA7 3′UTR fragment was cloned into the pGL3 plasmid Xbal I restriction site to construct the wild-type pgl3-wt-HOXA7 reporter plasmid. A mutated version of the pGL3 eukaryotic expression vector was constructed according to the miR-920 binding site on the HOXA7 3′UTR as directed by the GeneTailor Site-Directed Mutagenesis System kit. Human bone marrow mesenchymal stem cells were transfected into the following four groups: a HOXA7 wild-type plasmid control group (wild-type HOXA7 plasmid and miRNA mimics) and experimental group (wild-type HOXA7 plasmid and miR-195 mimics) and a HOXA7 mutant plasmid control group (mutant HOXA7 plasmid and miRNA mimics) and experimental group (mutant HOXA7 plasmid and miR-920 mimics). Cells were collected 24 h after transfection, and a Luciferase Reporter System was used to detect luciferase activity in each group. Relative luciferase activity was measured by the ratio of reporter (firefly) to control (Renilla) activity.

### Main observation indicators

The expression of miR-920, HOXA7, ALP, and OSX and alkaline phosphatase activity during osteogenic differentiation of bone marrow mesenchymal stem cells was measured in cells transfected with miR-920 mimic, inhibitor, and negative control.

### Statistical analysis

The SPSS 18.0 statistical software was used for statistical data analysis. Measurement data are expressed as the mean ± standard deviation, and a *t* test was used to compare the differences between groups. *P* < 0.05 was considered significant.

## Results

### MiR-920 is downregulated in OP patients

**Fig. 1:**
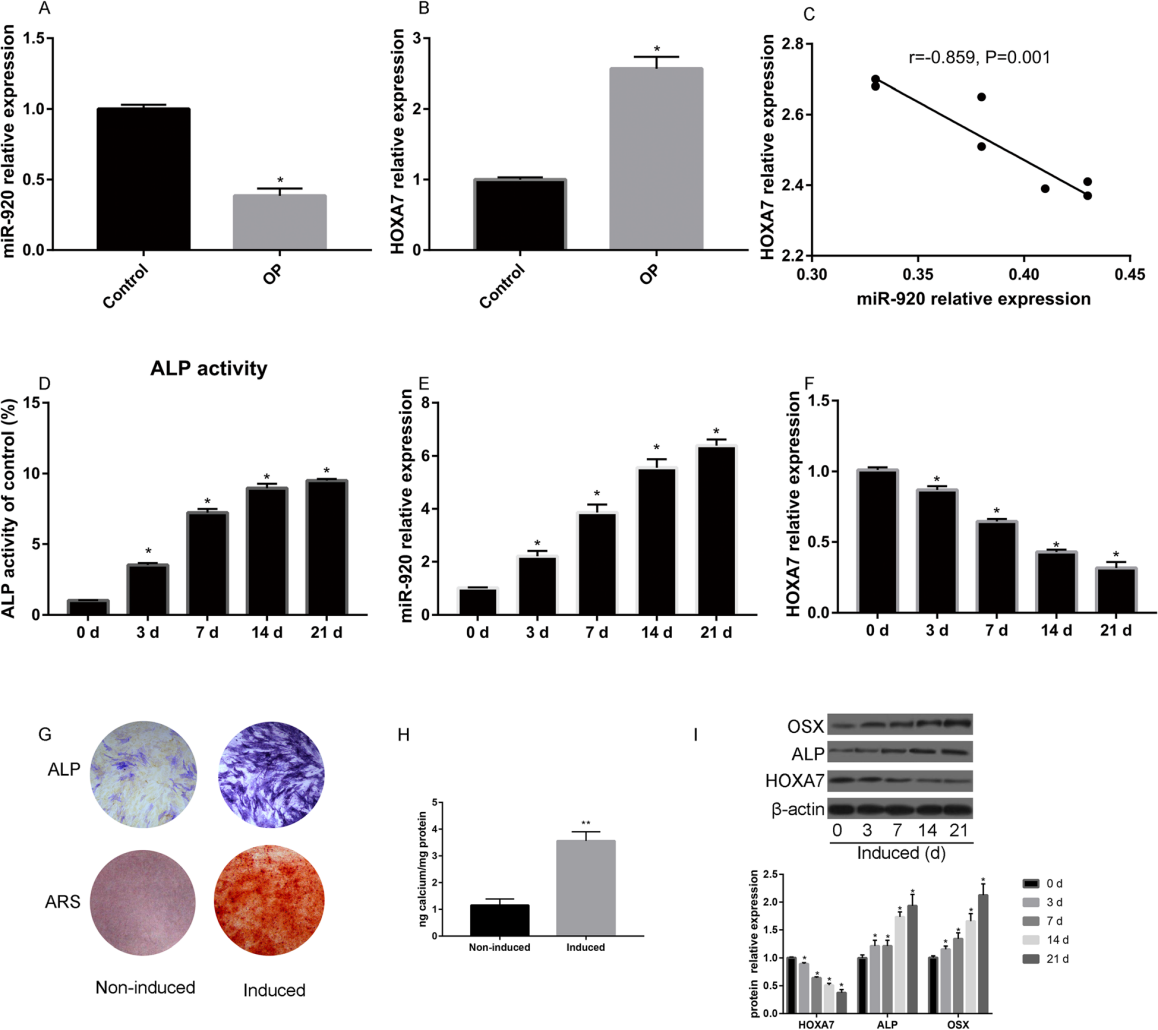
Relative expression of miR-920 (**a**) and HOXA7 (**b**) in control and osteoporosis patients; **c** correlation analysis of miR-920 and HOXA7; **d** ALP activity change during osteogenic differentiation; miR-920 (**e**) and HOXA7 (**f**) expression in the process of osteogenic differentiation of hBMSCs; **g** ALP and ARS in the noninduced and induced groups; **h** quantitative analysis of ARS staining in the noninduced and induced groups; **i** relative HOXA7, ALP, and OSX protein expression and quantitative analysis during osteogenic differentiation of hBMSCs

As illustrated in Fig. 1a, compared with the control, OP patients had reduced miR-920 levels (*P* < 0.05) and increased relative expression of HOXA7 (*P* < 0.05, Fig. 1b). Moreover, miR-920 had a negative correlation with HOXA7 (*r* = − 0.859, *P* = 0.001, Fig. 1c).

### MiR-920 is increased during osteogenic differentiation

As shown in Fig. 1d, the ALP activity increased as the induction time increased. In addition, we also found that miR-920 was increased during osteogenic differentiation (Fig. 1e). HOXA7 was decreased as the induction time increased (Fig. 1f). ALP and ARS results also showed that the induced group had higher ALP activity and calcium deposition than the control group (Fig. 1g). Western blot results showed that as the induction time increased, the relative expression of ALP and OSX increased compared with that at the beginning of induction (Fig. 1h, i).

### Bioinformatic analysis of miR-920

**Fig. 2:**
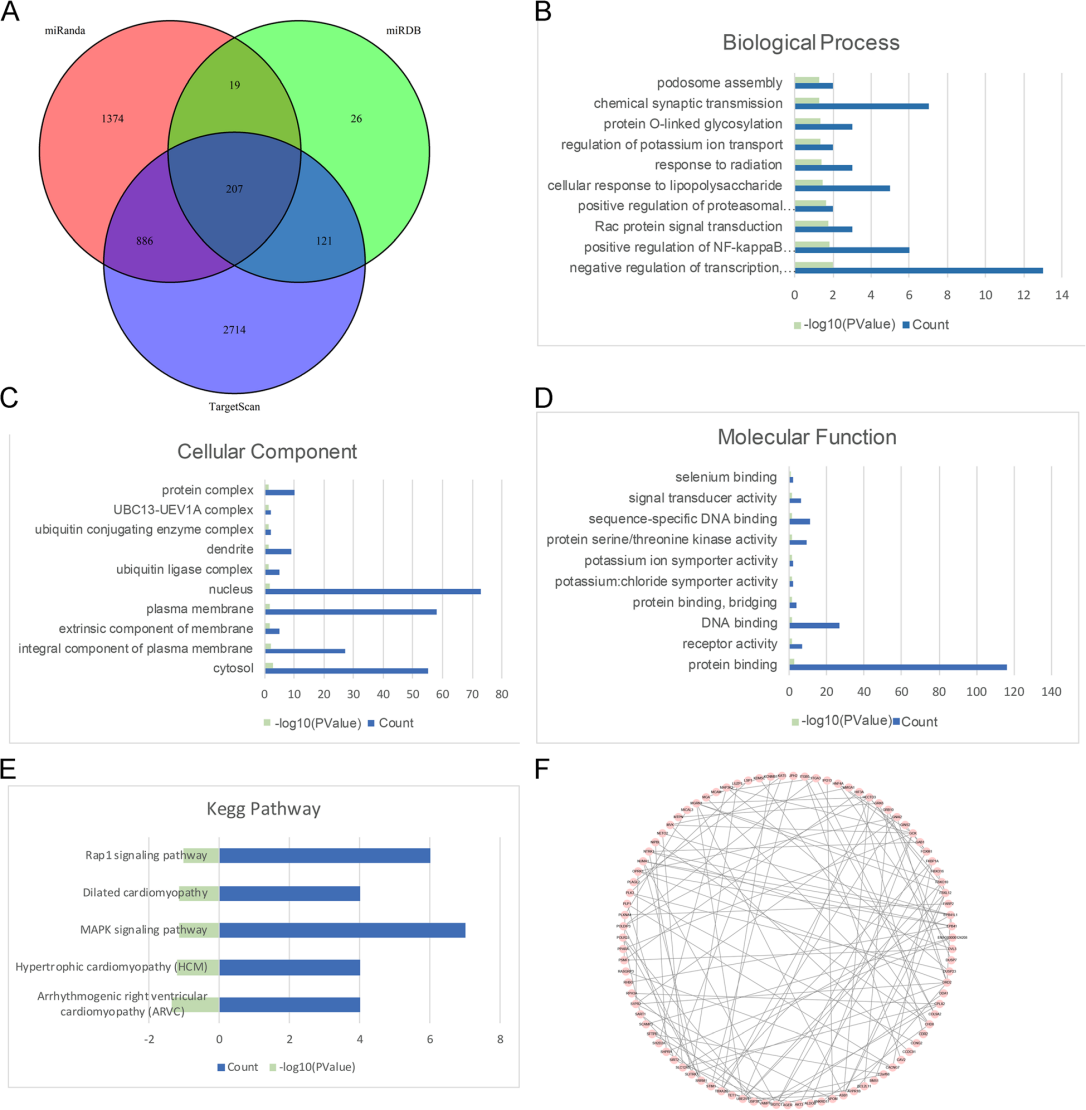
**a** Venn diagram of the overlapping genes of miR-920; **b** Gene Ontology analysis (biological process (**b**), cellular component (**c**), and molecular function (**d**)) of the target genes; **e** KEGG pathway of the target genes; **f** protein-protein interaction of the target genes

First, the miRanda, miRDB, and TargetScan databases were used to identify the overlapping genes targeted by miR-920. A Venn diagram revealed that there were a total of 207 overlapping genes (Fig. 2a). Figure 2 b, c, and d present the biological process, cellular component, and molecular function of miR-920. Figure 2 e presents the KEGG pathway of miR-920. We found that the target genes were mainly enriched in the MAPK signaling pathway. Figure 2 f shows the PPI network of the target genes identified through the STRING database.

### MiR-920 promotes osteogenesis of hBMSCs through the HOXA7-mediated MAPK signaling pathway

**Fig. 3:**
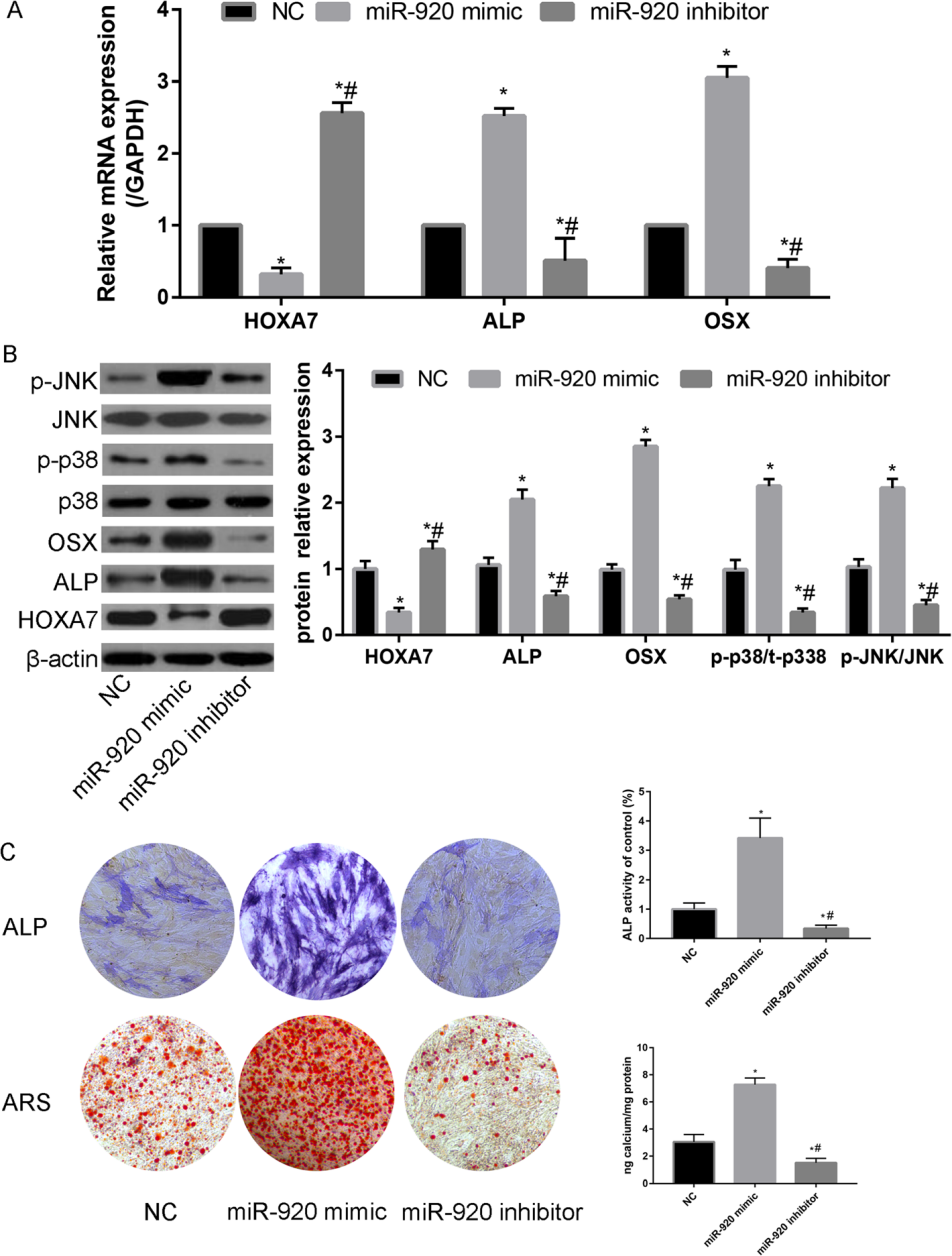
**a** Relative mRNA expression of HOXA7, ALP, and OSX in the NC, miR-920 mimic, and miR-920 inhibitor groups; **b** HOXA7, osteoblastic protein (ALP and OSX), p38, p-p38, JNK, and p-pJNK protein expression and quantitative analyses in the NC, miR-920 mimic, and miR-920 inhibitor groups; **c** ALP and ARS staining and quantitative analyses in the NC, miR-920 mimic, and miR-920 inhibitor groups

Figure 3 a demonstrates that compared with the NC group, miR-920 significantly downregulated HOXA7. Transfection of miR-920 mimic was associated with an increase in osteogenic differentiation-related proteins (ALP and OSX). We further explored the potential mechanism of miR-920 in regulating the osteogenic differentiation of hBMSCs. We found that miR-920 significantly activates p-p38 and p-JNK expression.

### MiR-920 directly targets HOXA7

**Fig. 4:**
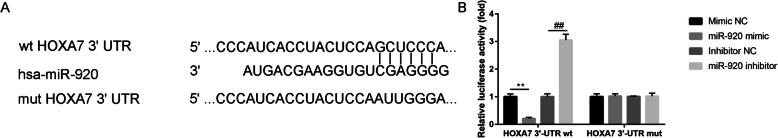
**a** Target sites of miR-920 and HOXA7; **b** luciferase activity in each group

The target gene of miR-920 was predicted by the TargetScan online database, and HOXA7 was found to be a potential target of miR-920. The 3′UTR of HOXA7 mRNA and the seed region of miR-920 had a theoretical complementary base pair sequence (Fig. 4a). Luciferase experiments were performed, and the results showed that the luciferase activity of the cotransfected HOXA7 wild-type group was significantly lower than that of the control group, while the luciferase activities of the cotransfected HOXA7 mutant group and the control group were not significantly different, which confirmed that miR-920 could directly act on HOXA7 (Fig. 4b).

## Discussion

The bone is a continuous and dynamically balanced tissue that mainly consists of two types of cells, osteoblasts, and osteoclasts [12]. Osteoblasts promote bone formation, and osteoclasts promote bone resorption [13, 14]. These cell types are balanced in normal bone tissue, but once this balance is broken, various kinds of bone metabolic diseases occur.

HBMSCs are adult stem cells with multidirectional differentiation ability that can be induced into osteoblasts, chondroblasts, adipocytes, and other cells under different conditions [15, 16]. Improving the osteogenic differentiation ability of bone marrow mesenchymal stem cells is the key to bone regeneration [17].

MiRNA is a small RNA with recently discovered regulatory functions that plays an important regulatory role in various biological processes [18]. An increasing number of miRNAs have been reported to regulate the osteogenic differentiation of bone marrow mesenchymal stem cells. Wang et al. [19] reported that the overexpression of miR-346 promotes the differentiation of bone marrow mesenchymal stem cells into osteoblasts by targeting GSK3b to regulate the Wnt signaling pathway. Zhang et al. [20] found that the expression of miR-20a increased gradually during the differentiation of bone marrow mesenchymal stem cells into osteoblasts. By studying the molecular mechanism of miR-20a regulating osteogenic differentiation, miR-20a was found to promote osteogenic differentiation of bone marrow mesenchymal stem cells through the regulation of the BMP/Runx2 signaling pathway by targeting PPAR gamma, Bambi, and Crim1. Zeng et al. [21] reported that miR-29b promotes osteogenic differentiation by directly downregulating the proteins HDAC4, TGF beta 3, ACVR2A, CTNNBIP1, and DUSP2, which are inhibitors of osteogenic differentiation.

We first determined the relative expression of miR-920 in OP and healthy controls. We found that miR-920 was decreased in OP patients, and miR-920 had a negative correlation with HOXA7. Zhou et al. [22] first found that miR-920 was involved in the pathogenesis of gouty arthritis. There have been no reports on the role and mechanism of miR-920 in regulating the osteogenic differentiation of hBMSCs. Then, we used OIM to induce osteogenic differentiation of hBMSCs. We found that miR-920 was increased during osteogenic induction of hBMSCs. Bioinformatic analysis is a novel technique to identify the potential target genes of miR-920. To increase credibility, three databases were used to identify a total of 207 overlapping target genes. Further analysis revealed that miR-920 potentially targets the HOXA7-mediated MAPK signaling pathway. These predictions were verified by KEGG pathway analysis, and western blotting was used to analyze p38, p-p38, JNK, and p-JNK expression.

Next, liposome transfection was performed to generate hBMSCs with low expression and high expression of miR-920 for osteogenic differentiation studies. By detecting alkaline phosphatase activity and the expression of OSX and ALP, markers of bone differentiation, miR-920 was found to promote osteogenic differentiation of hBMSCs.

Subsequently, a luciferase assay confirmed that HOXA7 is indeed a direct target of miR-920. In addition, the expression level of HOXA7 protein in hBMSCs transfected with miR-920 mimic was detected, and it was found that the transfected miR-920 inhibitor significantly increased the expression level of HOXA7 protein, which further confirms the direct effect of miR-920 on HOXA7. A study by da Silva et al. [23] demonstrated that HOXA cluster gene expression was increased during osteoblast differentiation, which is consistent with our conclusion. Previous studies demonstrated that the HOXA gene cluster is generally recognized as a pivotal mediator of positional identity in the skeletal system. The MAPK signaling pathway is crucial for osteogenic differentiation of hBMSCs. We used bioinformatic analysis and found that miR-920 promotes osteoblast differentiation potentially through the MAPK signaling pathway.

## Conclusion

**Fig. 5:**
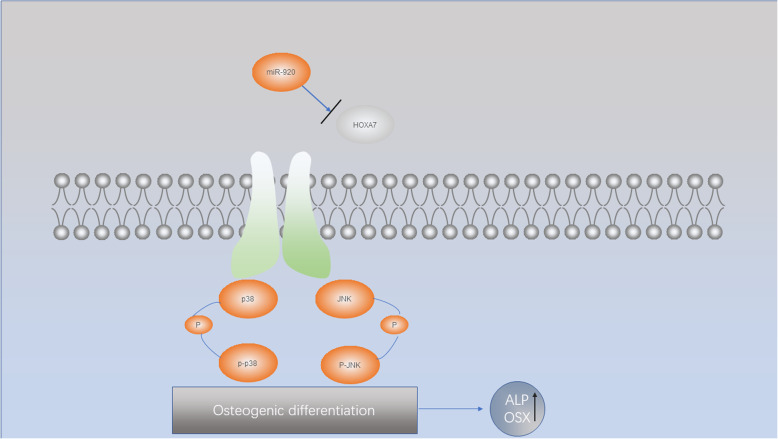
A brief schematic diagram showing the underlying mechanism by which miR-920 promotes osteogenic differentiation of hBMSCs

In conclusion, we first determined that miR-920 promotes the osteogenic differentiation of hBMSCs through the HOXA7-mediated MAPK signaling pathway (Fig. 5). Further studies should be focused on the role of miR-920 in osteogenesis in vivo.

## Data Availability

We declare that the materials described in the manuscript will be freely available to all scientists for noncommercial purposes.

## Abbreviations

hBMSCs: Human bone mesenchymal stem cells
miRNAs: MicroRNAs
FBS: Fetal bovine serum
